# Dextrose Prolotherapy's Impact on the Urinary Microbiome in Interstitial Cystitis/Bladder Pain Syndrome

**DOI:** 10.7150/ijms.104028

**Published:** 2025-02-26

**Authors:** Liang-Kun Chen, Shu-Jen Chang, Chin-Li Chen, Jing-Heng Yan, Juin-Hong Cherng, Gang-Yi Fan, En Meng, Yi-Chiung Hsu

**Affiliations:** 1 Department of Biomedical Sciences and Engineering, National Central University, Taoyuan City, Taiwan R.O.C.; 2 Institute of Biology and Anatomy, National Defense Medical Center, Taipei City, Taiwan R.O.C.; 3 Division of Urology, Department of Surgery, Tri-Service General Hospital, National Defense Medical Center, Taipei City, Taiwan R.O.C.; 4 Graduate Institute of Medical Sciences, National Defense Medical Center, Taipei City, Taiwan R.O.C.; 5 Department of Urology, SongShan Branch, Tri-Service General Hospital, National Defense Medical Center, Taipei City, Taiwan R.O.C.; 6 Graduate Institute of Life Sciences, National Defense Medical Center, Taipei 114, Taiwan R.O.C.; 7 Department of Biomedical Engineering, Chung Yuan Christian University, Taoyuan 320, Taiwan R.O.C.; 8 Department and Graduate Institute of Biochemistry, National Defense Medical Center, Taipei City, Taiwan R.O.C.; 9 Center for Astronautical Physics and Engineering, National Central University, Taoyuan, 320, Taiwan R.O.C.; 10 Department of Medical Research, Cathay General Hospital, Taipei, Taiwan R.O.C.

**Keywords:** 16S sequencing, interstitial cystitis, bladder pain syndrome, microbiome, urinary microbiome

## Abstract

**Background**: Interstitial cystitis/bladder pain syndrome (IC/BPS) is a debilitating chronic condition affecting millions globally. Dextrose prolotherapy, a minimally invasive and safe treatment, has emerged as a potential way to promote tissue healing in these patients. This study investigates how dextrose prolotherapy impacts the urinary microbiome, aiming to uncover the underlying mechanisms involved in its effectiveness.

**Methods:** Midstream urine samples from healthy controls and IC/BPS patients were collected before and after administering intravesical 10% dextrose injections. Microbiome profiling was conducted using 16S rRNA gene sequencing to analyze bacterial composition.

**Results:** Significant differences in urinary microbiome diversity were observed between healthy controls and IC/BPS patients. Proteobacteria, Firmicutes, and Bacteroidota were more abundant in IC/BPS patients. Importantly, dextrose prolotherapy led to a decrease in harmful bacteria (Subgroup_22, *Chryseolinea, and Ureaplasma*) while enriching beneficial species such as *Luteolibacter*, *Lactococcus*, and* L. lactis*, correlating with improved clinical symptoms.

**Conclusions:** Dextrose prolotherapy (DP) not only reduces the presence of harmful bacteria but also fosters the growth of beneficial microbes in IC/BPS patients. These findings suggest that the modulation of the urinary microbiome may be a key factor in its therapeutic success.

## Introduction

Interstitial cystitis/bladder pain syndrome (IC/BPS) is a chronic disease that affects the bladder or urethra, disrupting urinary processes and causing unpleasant pelvic pain.^1^ The major cause of IC/BPS remains unclear, and it is associated with unknown specific identifiable triggers. There are many theories have emerged to explain the pathogenicity of IC/BPS, including mast cell activation, neurogenic inflammation, urinary tract infection, tight junction protein downregulation, afferent nerve plasticity, bladder epithelial dysfunction, urothelial signaling disturbances, autoimmune disorders, high urothelium permeability, pelvic floor dysfunction, and psychosomatic factor.^2^ Although the clinical characteristics of IC/BPS are largely unknown, there are accumulated evidences on possible theories for its etiology, pathophysiology, and epidemiology.^3^ This complexity leads to uncertain diagnoses, which consequently leads to ineffective treatment; therefore, the exploration of new and innovative treatments for IC/BPS is warranted. Recently, some treatments for IC/BPS have focused on restoring the glycosaminoglycan layer to control urothelial damage via intravesical instillation or prolotherapy.^4^ Prolotherapy aims to restore epithelial function, reduce inflammation, prevent neural activation, manage allergies, and relieve IC/BPS symptoms^5^ using chemicals such as hyaluronic acid, botulinum toxin A, dextrose, and heparin. Chen *et al.* demonstrated that DP is safe and enhances the stimulation of tissue remodeling in patients with IC/BPS.^6^ In addition, this therapy has been observed to be more beneficial than patients' prior intravesical hyaluronic acid and Botox treatments, which were administered at 6-month intervals but were not associated with improved patient condition.^7^ These promising outcomes suggest that further studies are necessary to evaluate the underlying mechanisms of DP in patients with IC/BPS.

Human microbiome is defined as a group of microbial genomes that help widen the human metagenome, which affects innate and adaptive immunity. Recent studies have demonstrated that microbiota have facilitated the understanding of disease etiology and helped in the diagnosis of diseases.^8^ The correlation between urinary tract microbiota and diseases, such as IC/BPS, can be determined using urine samples and advance technologies, including 16S rRNA high-throughput sequencing, metagenomic sequencing, and enhanced quantitative urine culture.^9^ Additionally, previous studies have demonstrated significant differences in the urinary microbiota between healthy individuals and patients with urologic disorders and IC.^10^, ^11^ Saha *et al.* systematically predicted significant biomarkers and possible signaling pathways involved in IC/BPS using an integrated analysis of Gene Expression Omnibus datasets, which demonstrated the differential expression of hub genes in IC/BPS patients.^12^^17^ However, the role of urinary microbiota in the etiology or symptoms of IC/BPS affected by treatment has not been clarified. The present study aimed to determine the effectiveness of DP in patients with IC/BPS by focusing on the urinary microenvironment. In addition, 16S rRNA high-throughput sequencing and bioinformatics analysis were performed to investigate the characteristics of the urinary microbiome in IC/BPS patients receiving dextrose treatment compared to those of healthy volunteers. Moreover, this study provides new insights into the etiology of IC/BPS at the genus- and species-level microbiome and the underlying mechanisms of DP for IC/BPS.

## Materials and methods

### Patients and controls

**Figure 1:**
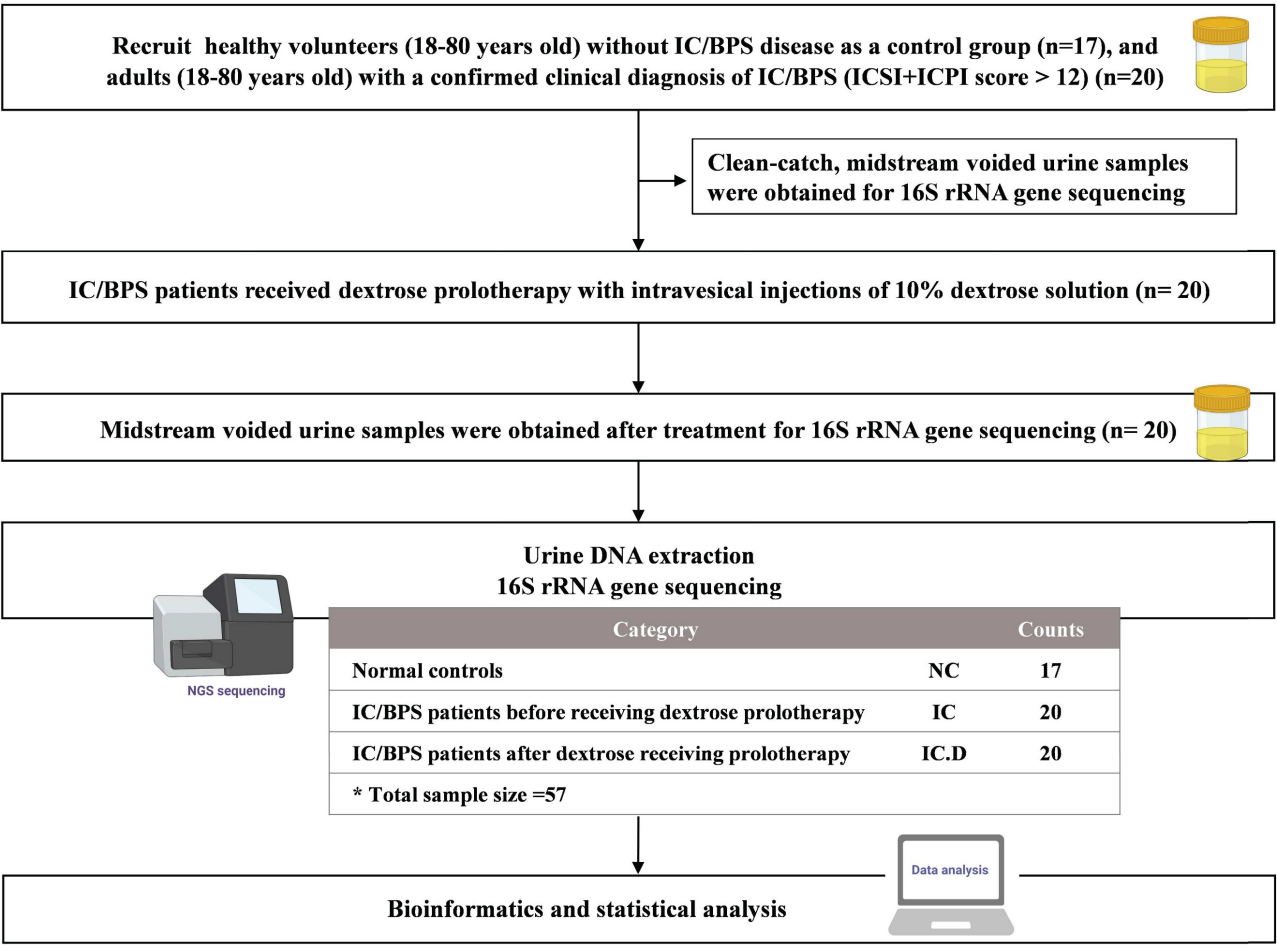
Flowchart of the study. In this flowchart, the yellow bottle drawing represents the collection of midstream voided urine for the examination of urinary microbiome.

Following the approval of the Institutional Review Board (IRB) of Tri-Service General Hospital for all phases of this project, the participants provided verbal and written consent for the collection and analysis of their urine for research purposes. Eligibility was defined as the absence of a known infection on clinical assessment (physician records and physical examination) and catheterized urine culture prior to surgery. Selected participants with a confirmed clinical diagnosis of IC/BPS were adults (age, 18-80 years) who were scheduled for treatment with DP under general anesthesia.^6^ The O'Leary-Sant instrument was used as a two-part certified symptom questionnaire (Interstitial Cystitis Symptom Index [ICSI] and Interstitial Cystitis Problem Index [ICPI]). The ICSI measures the severity and presence of typical IC/BPS symptoms, whereas the ICPI evaluates the level of patient discomfort caused by these symptoms. Each section comprises four questions, with the ICSI section having a response range of 0 (not at all) to 5 (nearly usually) and the ICPI section containing a response range of 0 (no problem) to 4 (major problem). The medical diagnosis of IC/BPS was confirmed by a total score (ICSI + ICPI) of > 12, with the greater numerical values suggesting an advanced stage of the disorder. For analysis in the present study, the patients were divided into three groups: IC/BPS patients before DP (IC group), IC/BPS patients after DP (IC.D group), and healthy volunteers (NC group). The NC group did not report any additional urinary symptoms or diagnoses. All participants provided a written informed consent and approval was obtained from the Institutional Review Board of our institution (IRB No. IRB1-107-05-192). Flowchart of the study is shown in Figure 1.

### Sample collection and preservation

Clean-catch, voided midstream urine samples were obtained with special emphasis on proper clinical collection techniques. Following in-clinic collection of urine specimens using standardized collection techniques, the specimens were preserved at 4 °C and processed for deoxyribonucleic acid (DNA) isolation within an hour. All specimens tested at the Tri-Service General Hospital were culture-negative. None of the patients were receiving antibiotics at the time of or before sample collection, according to hospital records.

Participants who provided informed consent and were compliant with the follow-up were recruited for the study. The study conformed to the ethical guidelines of the 1975 Declaration of Helsinki and was approved by the Institutional Review Board of the [IRB-1-107-05-192].

### Urinary DNA extraction and 16S rRNA gene amplification

Total genomic DNA was extracted from the samples using a QIAamp PowerFecal DNA Kit (Qiagen, Germany). Within 4 h of collection, 30 ml urine aliquots were centrifuged at 5,000 × g for 10 min, pelleted in lysis buffer (CD1), and subjected to two rounds of bead beating (6 m/s for 60 s, with a 5-min interval between cycles). The remaining aspects of the extraction protocol, involving multiple inhibitor removal and purification steps, were performed as described previously with one modification. Finally, DNA was eluted in 50 μl elution buffer (C6) and incubated at room temperature for 5 min; afterward, the eluent was passed through the silica membrane of the spin column a second time. DNA concentration was determined and adjusted to 5 ng/ul for the following process.

The 16S rRNA sequencing libraries were prepared according to the manufacturer's guidelines provided by Illumina (Illumina, CA, USA). Briefly, 12.5 ng of DNA was used for polymerase chain reaction (PCR) amplification of the V3 and V4 regions of the 16S rRNA gene. Afterwards, the PCR products were purified using AMPure XP beads (Beckman Coulter, USA) and subjected to a secondary PCR reaction with primers from the Nextera XT Index kit (Illumina, CA, USA) by adding dual indices and Illumina sequencing adapters. After PCR, the final libraries (approximately 630 bp) were purified using AMPure XP beads and were ready for next-generation sequencing.

### 16S rRNA gene amplicon sequencing

The concentrations of the 16S rRNA sequencing libraries were determined by real-time quantitative PCR using Illumina adapter-specific primers provided by the KAPA Library Quantification Kit (KAPA Biosystems, USA). Libraries were denatured and sequenced using an Illumina MiSeq platform with Reagent V3 for paired-end sequencing (2-300 bp). Instrument control, cluster generation, image capture, and base calling were performed using Real-Time Analysis (RTA) software, MiSeq Control software (MCS), and MiSeq Report software (MSR) on the MiSeq platform. The FASTQ files generated by the MiSeq Report were used for further analyses.

### Bioinformatics and statistical analysis

Raw reads were processed into amplicon sequence variants (ASVs) using the DADA2 pipeline in QIIME2^13^ to denoise, join the paired reads, and remove chimeras using default parameters. Afterward, the reads were reduplicated and ASVs were inferred. ASVs, which have unique DNA sequences, were separately assigned a taxonomy using the SILVA database (release 138).^14^ Taxa summaries were performed using QIIME2, whereas alpha rarefaction was performed using QIIME.^15^ Principal coordinate analyses (PCoA) were based on unweighted and weighted UniFrac distances and calculated using QIIME. Non-metric multidimensional scaling (NMDS) was analyzed using the vegan package (R package: v2.4 1)^16^ and heat maps were analyzed using the gplots^17^ package in R. High-dimensional biomarker discovery and explanation that recognizes genomics taxonomy characterizing the differences between two or more biological conditions were performed in Statistical Analysis of Metagenomic Profiles (STAMP).^18^ Differential analyses of the count data at the ASV level were applied to perform negative binomial generalized linear model implemented in DESeq2 R package.^19^ The functional content of 16S rRNA was predicted using PICRUSt software v2.2.0 b.^20^ Furthermore, differential analyses of functional content were performed using the negative binomial generalized linear model implemented in the DESeq2 R package. Statistical significance was obtained using one-way analysis of variance, post-hoc Tukey's test, receiver operating characteristic curve, Spearman correlation, and GraphPad Prism® version 9 (GraphPad Software, USA).

## Results

### Clinical characteristics of the participants

Due to the inability to collect urine from the bladder, the study investigated changes in the urinary microbiome by voided midstream urine. A total of 57 midstream urine specimens were collected from 17 participants of NC group, 20 participants of IC group, and 20 participants of IC.D group. These patients were the same 20 patients in both the IC and ICD groups. The clinical characteristics of the patients are shown in Supplementary Table 1. Only ICSI showed a significant difference between the IC and IC.D groups among several clinical disease indicators (*p* < 0.05).

### Urinary microbial diversity of healthy controls and IC/BPS patients

In total, 10,183,476 sequences were generated from midstream urine specimens collected from 57 participants (range: 113,350-453,240). After quality filtering, size filtering, and host sequence removal, 5,246,196 reads were clustered into ASVs (range, 45,088-200,336). Finally, 27,966 ASVs were generated for downstream analysis.

A rank-abundance curve was used to show the taxonomic richness and evenness of the samples. The number of ASVs was significantly higher in IC/BPS patients (n = 20) than in healthy controls (n = 17) [*p* < 0.0001], but not between the IC and IC.D groups (Supplementary Data S1 A). As measured by the Chao index (Supplementary Data S1 B) and Shannon index (Supplementary Data S1 C), urinary microbial diversity was significantly enhanced in the IC and IC.D groups compared to the NC group (*p* < 0.0001).

**Figure 2:**
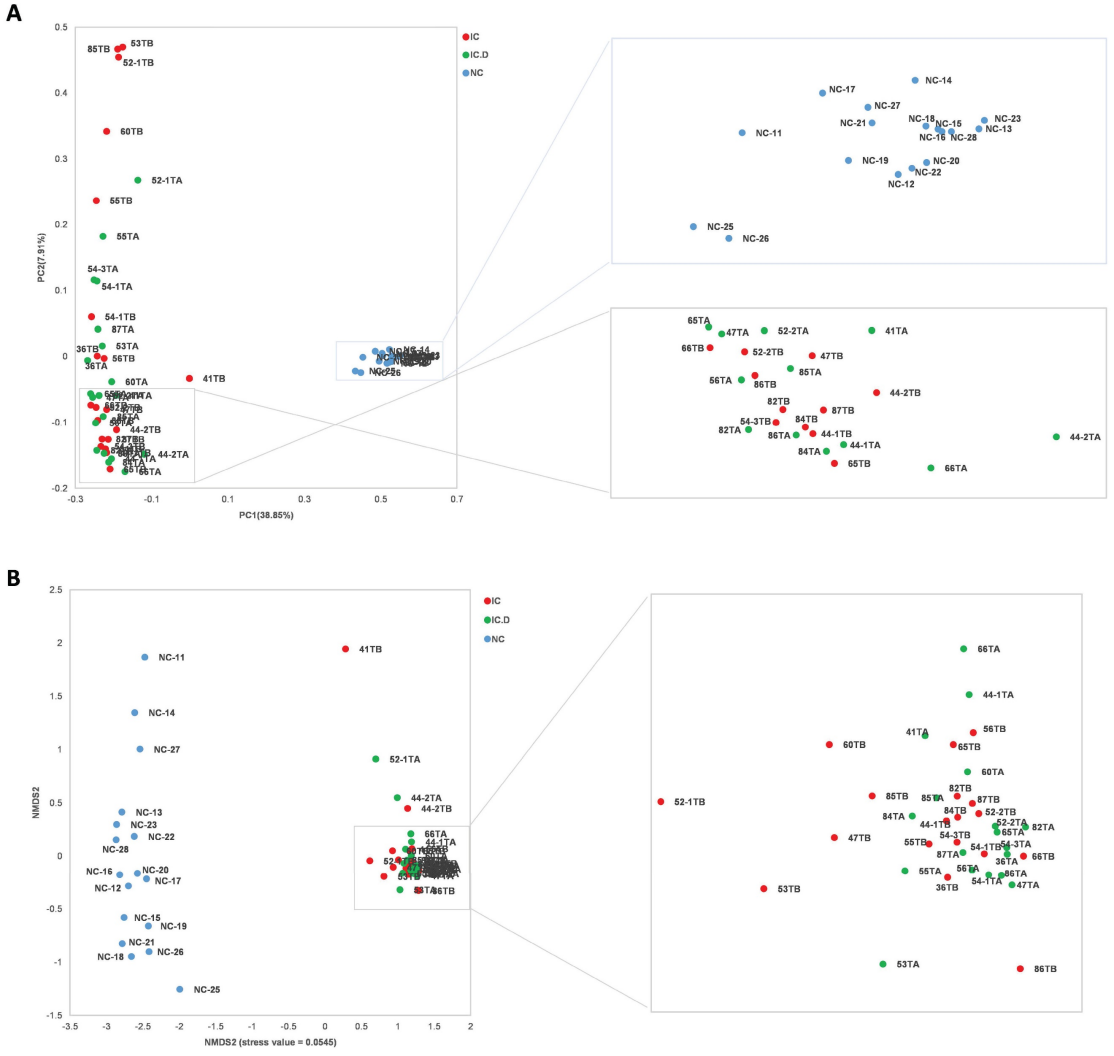
Urinary microbial diversity based on ASVs distribution in healthy controls and patients with IC/BPS (A) PCoA plot shows beta diversity based on the unweighted UniFrac distance. A distinct clustering of the samples was observed. The percentage of variation explained by PCoA1 and PCoA2 is noted along the axes. (B) NMDS plot showing beta diversity (stress value = 0.0545). The groups are distinguished by color. Each colored symbol corresponds to an individual sample. (PCoA, principal coordinate analysis; NMDS, non-metric multidimensional scaling).

In addition, PCoA and NMDS based on ASVs distribution were used to illustrate microbiome diversity. In the PCoA plot, the distribution of individuals in the IC group exhibited a long distance from the HC group and closely aligned with the IC.D group (Figure 2A). A similar result was found in the NMDS plot (Figure 2B). These results indicated that the urinary microbial composition was significantly different between patients with IC/BPS and healthy controls, while no significant differences were reported in the diversity of the microbiome of patients in the IC.D and IC groups.

### Urinary microbiome in healthy controls and IC/BPS patients

**Figure 3:**
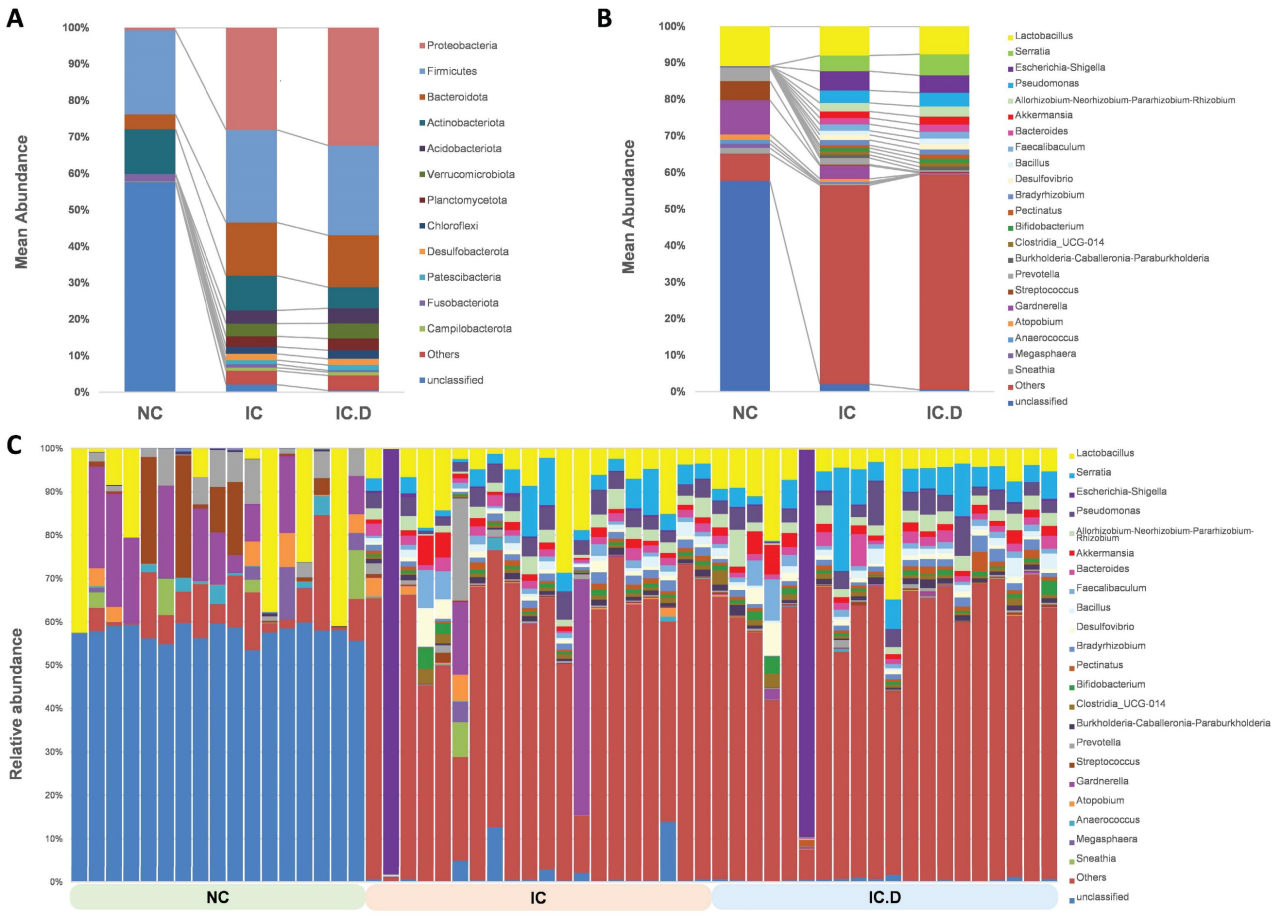
Urinary microbial composition of healthy controls and patients with IC/BPS Average composition and relative abundance of the bacterial community in the three groups at the (A) phylum and (B) genus levels (C) Differences in urinary microbial composition are shown in the NC, IC, and IC.D groups. Genera with an abundance of at least 1% in at least one group are shown.

Taxonomic analysis was performed using the average composition and relative abundance of the bacterial communities in the three groups at the phylum and genus levels (Figure 3A and 3B). Furthermore, the obtained data suggested that the abundances of *Actinobacteriota* and *Fusobacteriota* were higher in the NC group, whereas the most abundant phyla in the IC and IC.D groups were *Proteobacteria*, *Firmicutes* and *Bacteroidota*. Additionally, *Acidobacteriota*, *Verrucomicrobiota*, *Planctomycetota*, *Chloroflexi*, *Desulfobacterota*, and *Patescibacteria* were enhanced in the IC and IC.D groups but were deficient in the NC group (Figure 3A, Supplementary Table 2).

**Figure 4:**
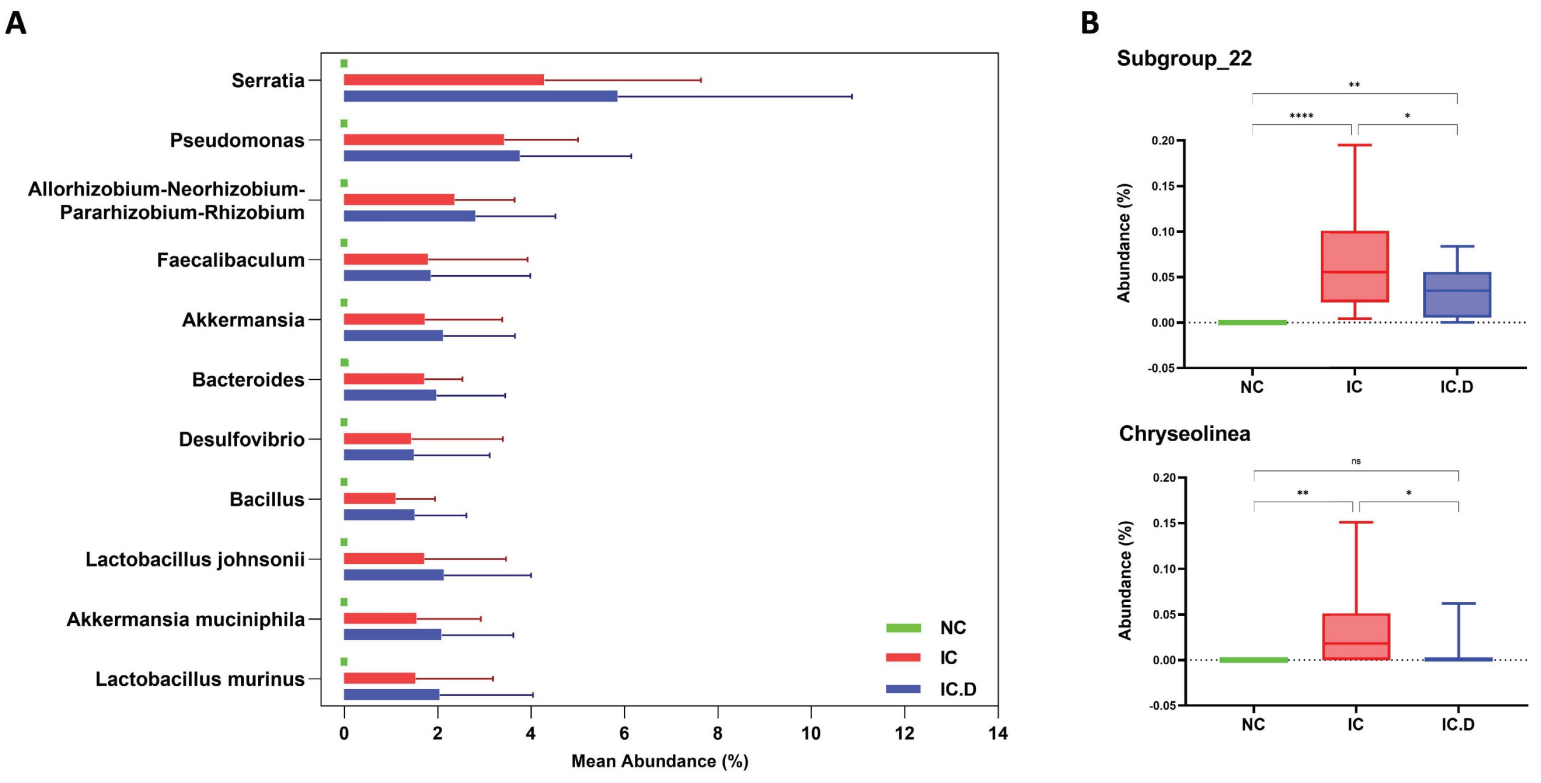
Bacteria that may play important roles in the pathogenesis of the disease. (A) Nine genera and three species were significantly enriched in the IC group, but were deficient in the NC group. (B) The two identified genera were significantly enriched in the IC group compared to the IC.D group, and their abundance decreased after dextrose prolotherapy, showing a restorative trend toward normal. *P*-values analyzed by one-way ANOVA with Tukey's post hoc test are shown for the genus Bacteria. (ns, not significant; ^*^p < 0.05, ^**^p < 0.01, ^***^p < 0.001, ^****^p < 0.0001; ANOVA, analysis of variance).

*Lactobacillus* was the most common genus among patients with IC/BPS and NC. Furthermore, *Gardnerella* (9.33%), *Streptococcus* (5.22%), and *Prevotella* (3.76%) significantly increased in the NC group. The differences in the urinary microbial composition of each group are displayed in Figure 3C. Furthermore, *Lactobacillus*, *Serratia*, *Pseudomonas*, *Allorhizobium*-*Neorhizobium*-*Pararhizobium*-*Rhizobium*, *Akkermansia*, and *Bacteroides* were the most prevalent genera in the IC and IC.D groups. A similar tendency was observed at the genus level, in addition to the finding that some phyla were abundant in the IC group but not in the NC group (Supplementary Table 2). These bacteria were selected based on STAMP analysis, which focused on bacteria that were significantly more prevalent in the IC group than in the NC group. Moreover, they were selected based on the following criteria: an average abundance of at least 1% in the IC group, the prevalence of at least 50%, and deficiency in the NC group. Subsequently, eight genera (*Serratia*, *Pseudomonas*, *Allorhizobium*-*Neorhizobium*-*Pararhizobium*-*Rhizobium*, *Faecalibaculum*, *Akkermansia*, *Bacteroides*, *Desulfovibrio*, and *Bacillus*) and three species (*Lactobacillus johnsonii*, *Akkermansia muciniphila*, and *Lactobacillus murinus* (synonymous with *Ligilactobacillus murinus*)) were selected as the bacteria that may play important roles in the pathogenesis of the disease (Figure 4A). Furthermore, in comparing the microbial composition before and after DP in both groups, two genera, *Subgroup_22* and *Chryseolinea*, were significantly enhanced in the IC group than in the IC.D group. However, their abundance decreased after treatment, showing a restorative trend towards the microbial composition observed in the NC group. Additionally, these genera were initially deficient in the NC group, suggesting their potential as pathogens affected by DP (Figure 4B).

### Correlation between the urinary microbiome and clinical index of IC/BPS patients

**Figure 5:**
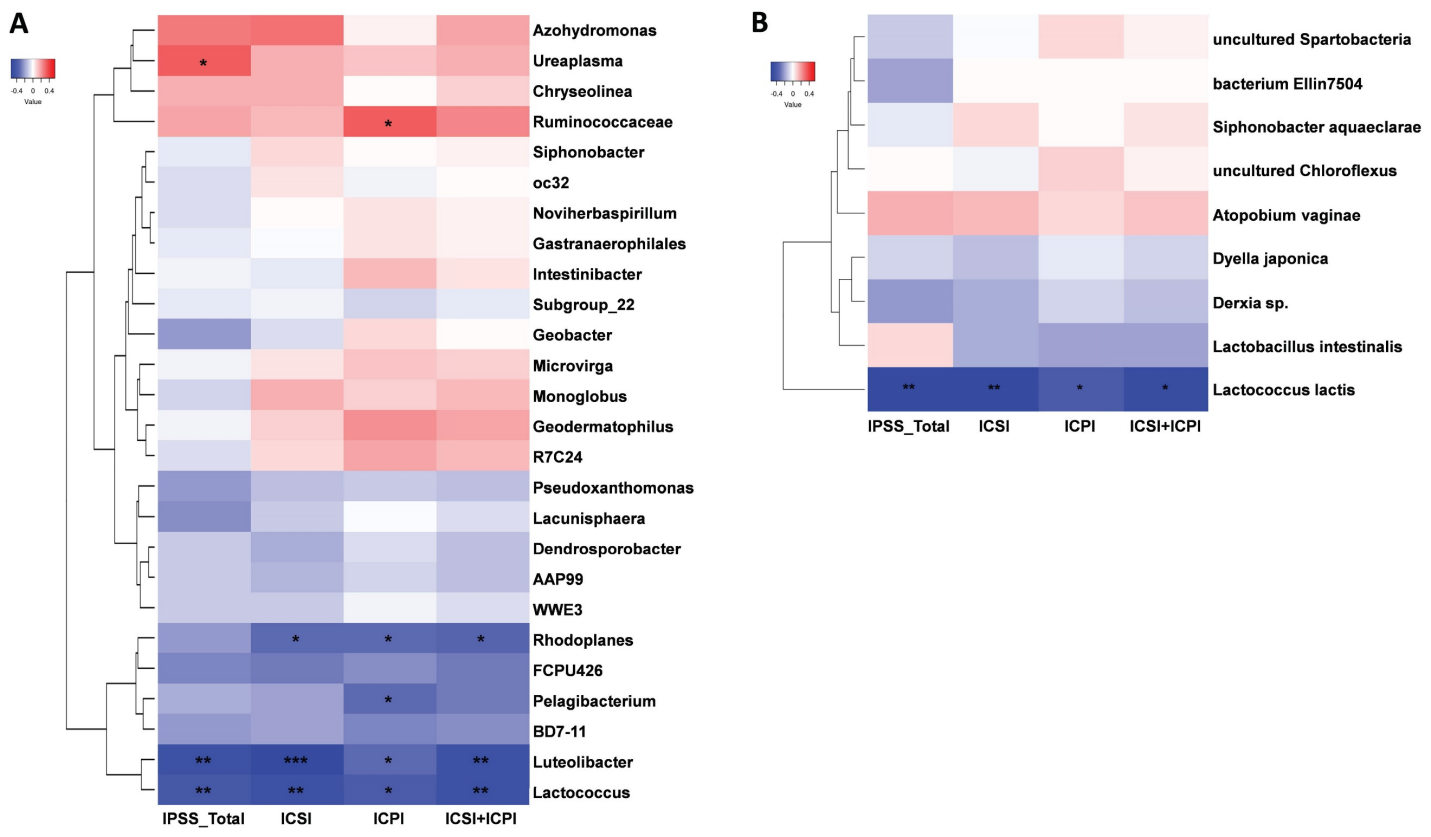
Heatmap of Spearman's rank correlation coefficients between significantly different bacteria and clinical indices at the (A) genus and (B) species level. Red box = positive correlation; blue box = negative correlation. (^*^p < 0.05, ^**^p < 0.01, ^***^p < 0.001, ^****^p < 0.0001).

The effects of DP in patients with IC/BPS were revealed by the use of STAMP analysis to compare the urinary microbiome of the IC and IC.D groups. The results showed that four genera and one species were significantly enriched in the IC group, whereas the IC.D group exhibited an enrichment of 22 genera and eight species (Supplementary Data S2). Spearman's rank correlation was performed to further understand the correlation between the bacteria that exhibited significant differences in abundance due to DP and clinical indicators. The heatmaps in Figure 5 were constructed based on Spearman's rank correlation values. For the heatmaps, we used Euclidean distance as the similarity metric, average linkage as the clustering method, and normalized the data using Z-score normalization to show better color contrast. At the genus level, *Ureaplasma*, *Ruminococcaceae*, *Rhodoplanes*, *Pelagibacterium*, *Luteolibacter*, and *Lactococcus* were significantly correlated with the clinical indices (Figure 5A, Supplementary Table 3). *Ureaplasma* enriched in the IC group showed a significant moderate positive correlation with the total IPSS index. *Luteolibacter*, and *Lactococcus* enriched in the IC.D group showed a significant moderate negative correlation with all four clinical indices. Moreover, at the species level, *Lactococcus lactis* exhibited a significant moderate negative correlation with the clinical indices (Figure 5B, Supplementary Table 4). Notably, *L. lactis* was also enriched in the IC.D group.

### Important microbial functions related to disease and DP

PICRUSt was used to predict the relative abundance of functional genes based on the obtained 16S rRNA sequences and determine the abundance and enrichment of functional genes at different levels in the Kyoto Encyclopedia of Genes and Genomes (KEGG) pathways.

**Figure 6:**
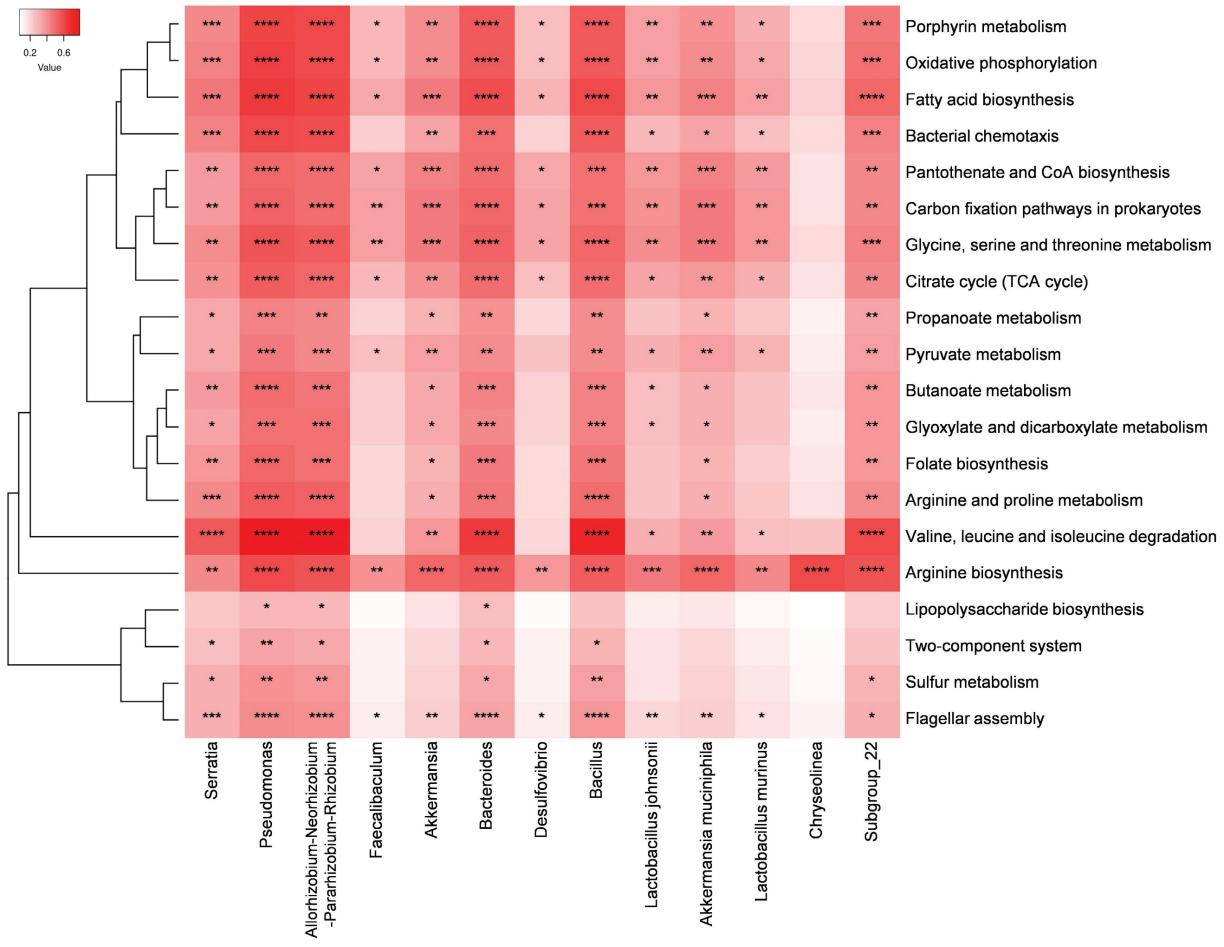
Heatmap of Spearman's rank correlation coefficients between the 13 bacteria that may play important roles in the pathogenesis of the disease and the top 20 significantly enriched pathways in the IC group compared to those in the NC group. Red box = positive correlation. (^*^p < 0.05, ^**^p < 0.01, ^***^p < 0.001, ^****^p < 0.0001).

STAMP analysis revealed that over 300 metabolic pathways exhibited significant differences in abundance between the IC and NC groups. Subsequently, the top 20 pathways enriched in the IC group and 13 bacteria that may play important roles in the pathogenesis of the disease were selected for Spearman's rank correlation analysis. *Pseudomonas*, *Allorhizobium*-*Neorhizobium*-*Pararhizobium*-*Rhizobium*, and *Bacteroides* were significantly correlated with these 20 pathways and exhibited a strong positive correlation with the valine, leucine, and isoleucine degradation pathways (Figure 6). Moreover, *Serratia*, *Bacillus*, and *Subgroup_22* were significantly correlated with 18 of these pathways. Additionally, the arginine biosynthesis pathway was significantly and positively correlated with all 13 potential pathogens.

**Figure 7:**
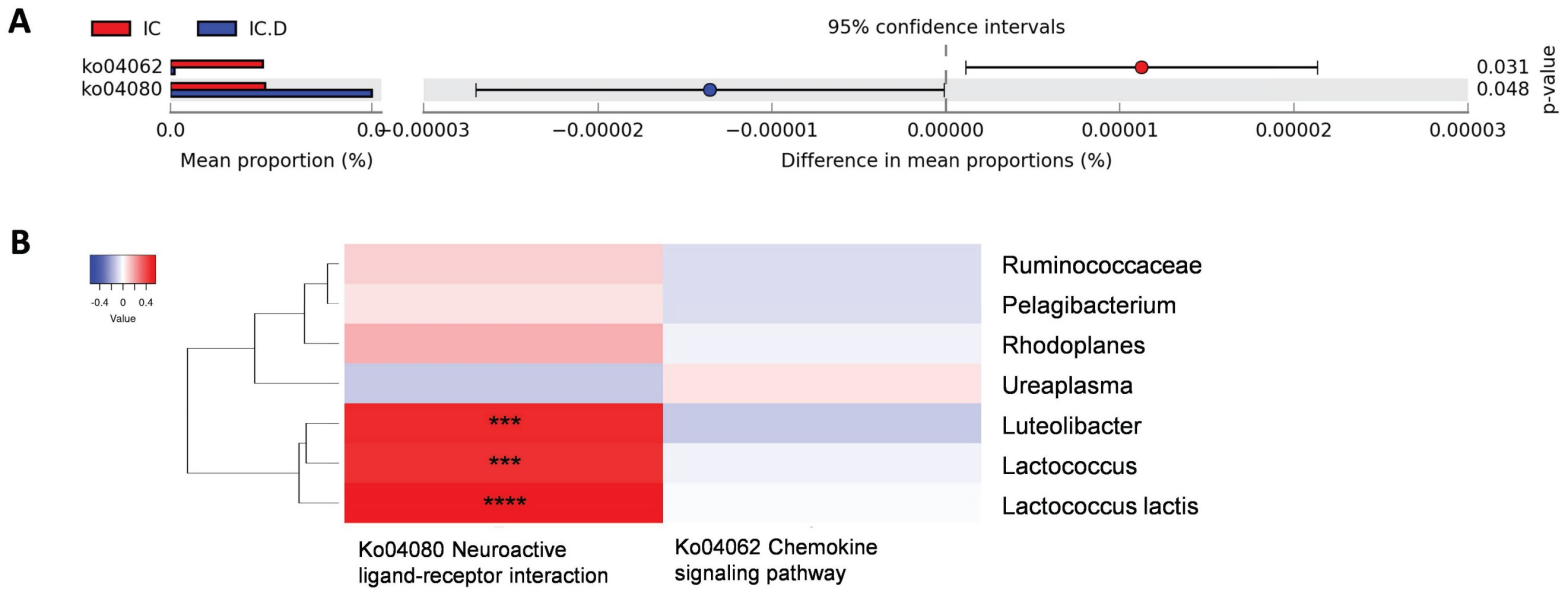
Crucial microbial functions related to dextrose prolotherapy. (A) STAMP analysis revealed pathways with significant differential abundance (*p* < 0.05) between the IC and IC.D groups. (B) Heatmap of Spearman's rank correlation coefficients between pathways with significant differential abundance and significantly different bacteria between the IC and the IC.D groups. Red box = positive correlation; blue box= negative correlation. (^*^p < 0.05, ^**^p < 0.01, ^***^p < 0.001, ^****^p < 0.0001).

The pathways that showed significant differences before and after DP were neuroactive ligand-receptor interactions enriched in the IC.D group and the chemokine signaling pathway enriched in the IC group (Figure 7A). To measure the correlation between these two pathways and previously identified bacteria related to clinical indices, Spearman's rank correlation analysis was performed. The results showed a significant and moderately positive correlation between *Luteolibacter*, *Lactococcus*, and *L. lactis* and the neuroactive ligand-receptor interaction pathway (Figure 7B).

## Discussion

Our study revealed distinct differences in the urinary microbiome between healthy controls and IC/BPS patients. Several studies have reported inconclusive results regarding the diversity and genus-level composition of urinary microbiome in IC/BPS patients compared to healthy controls.^10^, ^21^, ^22^ In our study, we observed significantly higher urinary microbial diversity in IC/BPS patients than in healthy controls, accompanied by distinct compositional differences between IC patients and healthy controls. Zheng *et al.* also showed that *Lactobacillus* was the most abundant genus only in the control group, but not in the IC/BPS group, which differs from our findings. The results of this study indicate that *Lactobacillus* emerged as the most prevalent genus in IC, IC.D, and healthy control groups, consistent with previous research.^21^, ^22^ Furthermore, several studies reported that Gardnerella was the second most abundant genus in the urinary microbiome of NC.^22^, ^23^* Ralstonia*, *Bradyrhizobium*, *Sphingomonas*, *Bosea* and *Allorhizobium-Neorhizobium-Pararhizobium-Rhizobium* were reported to be significantly higher in the IC/BPS group,^10^ which is consistent with our findings. Allorhizobium-Neorhizobium-Pararhizobium exhibited an abundance of more than 1% and a prevalence of more than 50% in IC/BPS patients, while being deficient in the NC group. Interestingly, other bacteria with the most significant enriched and prevalent in IC/BPS group in our study have also been implicated in the pathogenicity of other diseases. For example, Serratia exhibited 100% prevalence in the IC group, and has been associated with infections in patients undergoing peritoneal dialysis.^24^ *Serratia* showed a significant positive correlation with metabolites such as tetradecylamine and phytosphingosine, which were elevated in IC/BPS patients.^25^ Phytosphingosine is a metabolite of the sphingolipid signaling pathway. The sphingolipid signaling pathway was significantly more abundant in the IC group than in the NC group; its upstream pathway, glycine, serine, and threonine metabolism pathway, was one of the top 20 pathways significantly abundant in the IC group. Moreover, *Serratia* showed a significant moderate positive correlation with glycine, serine, and threonine metabolism (r = 0.41, *p* = 0.0015).

Additionally, the presence of *Pseudomonas* was also evident, being an opportunistic pathogen. *P*. *aeruginosa* and *P*. *luteola* are pathogens that cause urinary tract infections.^26^ The study of infection with P. aeruginosa in mice lungs demonstrated that caused neuroinflammation, cerebral microvascular leakage and increased permeability of pericytes at the blood-brain barrier. The underlying mechanisms involve cytokine-induced disruption of cell-cell adhesion barrier function in brain endothelial cells and downregulation of cell-cell junctions.^27^ In the IC group, the abundance and prevalence of *Pseudomonas* were 3.42% and 100%, respectively, and this enriched *Pseudomonas* might potentially cause similar damage. In addition, substances generated by *Desulfovibrio* are generally involved in the pathogenesis of Parkinson's disease.^28^ Moreover, *Lactobacillus johnsonii* has been demonstrated to be present in greater amounts in IC patients than in healthy controls.^29^, ^30^

*Ureaplasma* is a bacterium without a cell wall, commonly found in the urinary and the most prevalent genital Mycoplasma isolated from genital tracts.^31^ *Ureaplasma* species are recognized as opportunistic pathogens in human genitourinary tract infections, infertility, adverse pregnancy, neonatal morbidities, and other adult invasive infections.^32^ In this study, the abundance of *Ureaplasma* showed a significant moderate positive correlation with the IPSS_total index, indicating that lower *Ureaplasma* abundance was associated with milder disease symptoms. After DP treatment, the abundance of *Ureaplasma* significantly decreased, suggesting that DP reduced harmful pathogens. Additionally, other studies showed that *Ureaplasma* affects the immune system. For instance, *U*. *parvum* induced a proinflammatory environment (cytokines) and increased MMP-9 in cervical epithelial cells.^33^ Furthermore, *Ureaplasma* spp. induced cell death and downregulated mRNA levels for proteins involved in inflammatory cell death suppression in human brain microvascular endothelial cells.^34^ DP treatment might improve symptoms by reducing the damage caused by *Ureaplasma* to the bladder endothelium.

Several studies have demonstrated that arginine metabolism regulates the pathogenesis of some diseases.^35^-^37^ In addition, in arginine biosynthesis pathway, arginase (Arg) metabolized arginine to urea and ornithine, and contributed to the pathogenesis of inflammatory bowel disease (IBD) and colitis. The expression of Arg1 was correlated with the higher inflammatory score in IBD patients.^36^ Furthermore, the expression of Arg1 was upregulated in both intestinal epithelial cells and myeloid cells,^36^ and the inhibition of Arg1 was effective for colitis treatment in a dextran sodium sulfate-induced colitis model in mice.^37^ The abundance of Arg was also enriched in the IC group. The result revealed the potential influence of the arginine biosynthesis pathway to the pathogenesis of IC/BPS.

DP was administered by injecting 10% dextrose into the bladder; however, this therapy also influenced the urinary microbiome. The comparative analysis of urinary microbiomes between IC and IC.D groups revealed that DP leaded to a significant increase in the abundance of certain bacteria. Interestingly, *Lactococcus lactis* (*L. lactis*) was the only species that correlated with clinical indexes and showed a significant moderate negative correlation with all four clinical indices, IPSS_total, ICSI, ICPI and ICSI+ICPI. This data demonstrated that the higher the level of L. lactis in the urine microbiome, the lower the degree of IC disease. Furthermore, the metabolic pathway of neuroactive ligand-receptor interaction was significantly enhanced in the IC.D group compared to the IC group, and this metabolic pathway showed a significant positive correlation with *L. lactis*. Furthermore, *L. lactis* is a non-pathogenic microorganism that is generally recognized as safe (GRAS) by regulatory agencies. *L. lactis* NCDO 2118 administration ameliorated colitis through immunomodulatory activity in mice, and was associated with an early increase in IL-6 production and sustained IL-10 production in colonic tissue.^38^ Additionally, the number of regulatory CD4+ T cells (Tregs) increased in mice fed *L. lactis* NCDO 2118.

In IC patients, a significant increase in IL-10 levels was detected, suggesting the upregulation of anti-inflammatory responses. Although the increase in IL-6 levels did not reach statistical significance, it was still noticeably elevated, suggesting its potential involvement in IC-related inflammatory processes.^6^ In contrast, *L. lactis* LL95 orally administrated to mice improved depressive- and anxiety-like behavior.^39^ Ma *et al.* found that the oral administration of *L. lactis* E001-B-8 in mice promoted the production of 5-HT (serotonin) and alleviated depressive and anxiety-like behaviors in response to chronic unforeseeable mild stress stimulation. These effects were associated with improvements in 5-HT metabolism and the modulation of gut microbioal composition.^40^ Because 5-HT is involved in the neuroactive ligand-receptor interaction pathway, it can be inferred that the promotion of 5-HT production by *L. lactis* also stimulates the activation of the neuroactive ligand-receptor interaction pathway. Accordingly, after DP, a notable increase in the abundance of this pathway was observed in IC patients. Although the prediction of functional profile using PICRUSt is sufficiently linked to phylogeny and provides useful insights, it does not provide direct evidence. Therefore, further studies are warranted to confirm the effects and mechanisms of changes in the urinary microbiome through metabolomic profiling. However, our study demonstrated that the abundance of *L. lactis* in the urinary microbiome was negatively associated with the severity of IC/BPS disease. This suggested that DR treatment fostered the growth of beneficial microbes in IC/BPS patients.

## Conclusions

Our study revealed distinct differences in the urinary microbiome between healthy controls and IC/BPS patients, with dextrose prolotherapy significantly impacting microbial composition. The therapy reduced potentially pathogenic bacteria and increased beneficial strains such as *Luteolibacter*, *Lactococcus*, and *L. lactis*. These findings suggested that the modulation of the urinary microbiome, potentially by enhancing the host immune system, may be a key factor in its therapeutic success.

## Supplementary Material

Supplementary figures and tables.

## Abbreviations

DP: dextrose prolotherapy
IC: Interstitial cystitis
IC/BPS: Interstitial cystitis/bladder pain syndrome
ICSI: Interstitial Cystitis Symptom Index
ICPI: Interstitial Cystitis Problem Index
PCoA: Principal coordinate analyses
NMDS: Non-metric multidimensional scaling
STAMP: Statistical Analysis of Metagenomic Profiles
