# Supplementary material for: Dextrose Prolotherapy's Impact on the Urinary Microbiome in Interstitial Cystitis/Bladder Pain Syndrome
Source: Int J Med Sci. 2025 Feb 26;22(7):1516–27. doi: 10.7150/ijms.104028 (PMC11905267; doi:10.7150/ijms.104028)
Supplement: Supplementary file 1 — Supplementary figures and tables. [file ijmsv22p1516s1.zip › Supplementary legends.pdf]

Supplementary data S1. Urinary microbial diversity in healthy controls and IC/BPS patients. Urinary microbial diversity was assessed using (A) the number of ASVs measured, (B) the Chao1 index, and (C) the Shannon index. Compared with the control group, the number of ASVs in the IC and IC.D groups increased significantly. *P*-values analyzed by one-way ANOVA with Tukey's post hoc test are shown for the genus Bacteria. (ns, not significant; $^{*}p < 0.05$, $^{**}p < 0.01$, $^{***}p < 0.001$, $^{****}p < 0.0001$; ANOVA: analysis of variance)

Supplementary data S2. STAMP analysis of the effects of dextrose prolotherapy on urinary microbiome of patients with IC/BPS. The analysis showed a significant difference ($p < 0.05$) between the two groups at the genus (A) and species (B) levels.

Supplementary table 1. Clinical characteristics in this study exhibited by patients with healthy controls (NC), IC/BPS patients before receiving dextrose prolotherapy (IC), and IC/BPS patients after receiving dextrose prolotherapy (IC.D). Categorical variables were compared using one-way ANOVA or Student's t-test.

Supplementary table 2. Phylum- and genus-level taxonomic succession of the urinary microbiome in healthy controls and patients with IC/BPS genera with an abundance of at least 1% in at least one group are shown.

Supplementary table 3. Spearman's rank correlation coefficients between significantly different bacteria and clinical indices at the genus level. Red box = positive correlation; blue box = negative correlation. (*p < 0.05, **p < 0.01, ***p < 0.001, ****p < 0.0001)

Supplementary table 4. Spearman's rank correlation coefficients between significantly different bacteria and clinical indices at the species level. Red box = positive correlation; blue box = negative correlation. (*p < 0.05, **p < 0.01, ***p < 0.001, ****p < 0.0001)
