# Supplementary figures and images for: Dextrose Prolotherapy's Impact on the Urinary Microbiome in Interstitial Cystitis/Bladder Pain Syndrome
Source: Int J Med Sci. 2025 Feb 26;22(7):1516–27. doi: 10.7150/ijms.104028 (PMC11905267; doi:10.7150/ijms.104028)

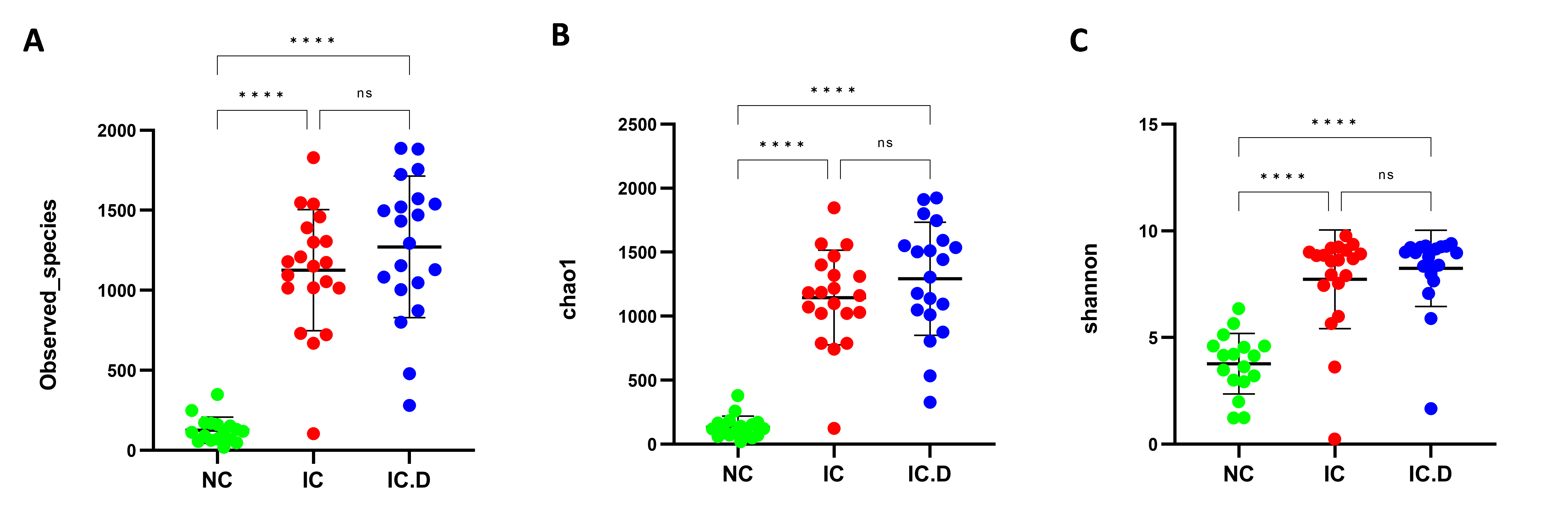

Supplement: Supplementary file 1 — Supplementary figures and tables. [file ijmsv22p1516s1.zip › Supplementary data S1.png]

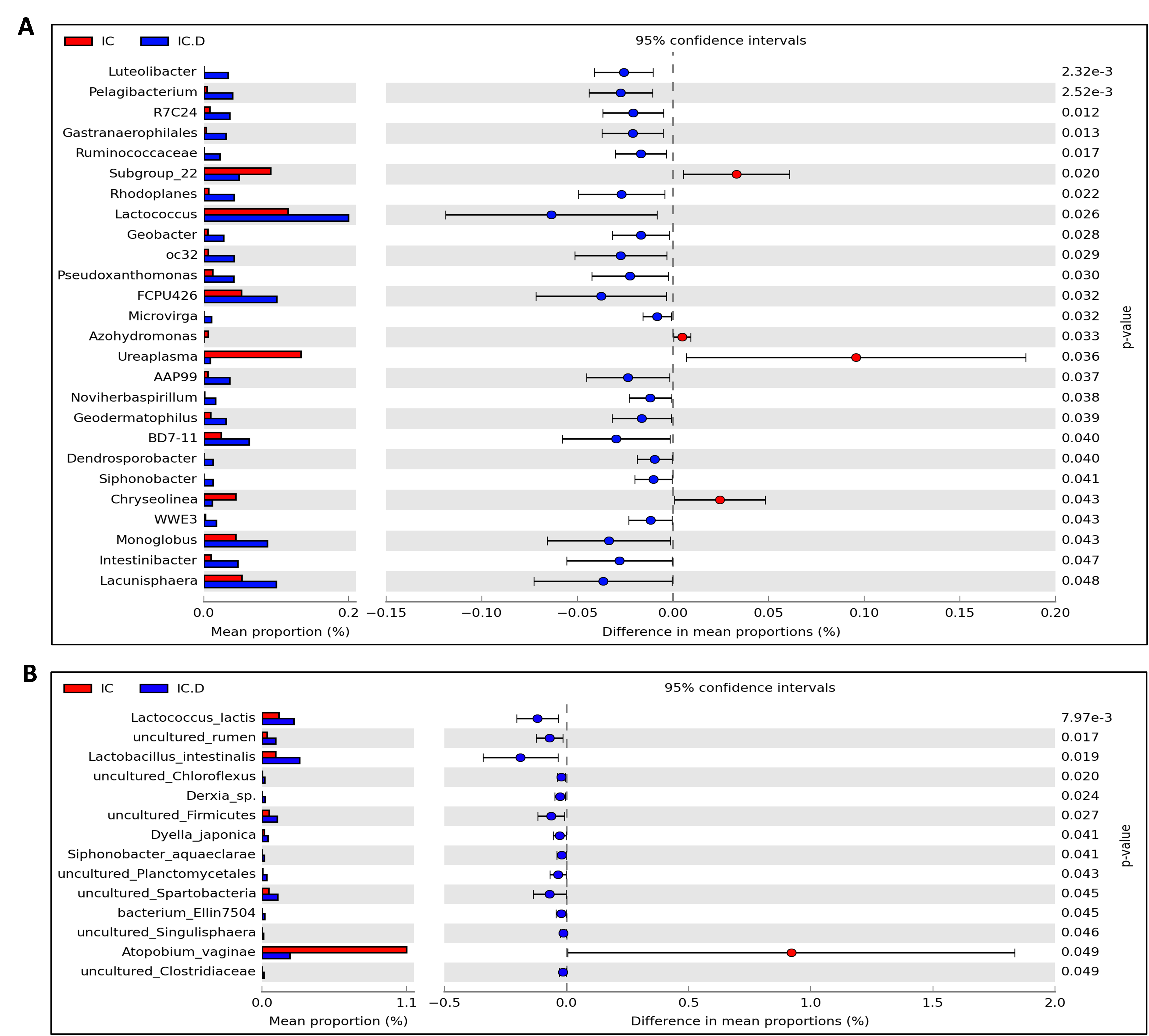

Supplement: Supplementary file 1 — Supplementary figures and tables. [file ijmsv22p1516s1.zip › Supplementary data S2.png]
